# Lippincott’s Magazine, an Illustrated Monthly of Popular Literature and Science

**Published:** 1873-03

**Authors:** 


*Lippincott's Magazine, an Illustrated Monthly of Popular Literature and Science.*

The March number of this superb illustrated monthly is on our table. *Lippincott's Magazine* ranks among the best and purest of our literary magazines. It is edited with great ability and profusely illustrated. J. B. Lippincott & Co., 715 and 717 Market street, Philadelphia. Price, $4.

